# The Future of Clinical Phage Therapy in the United Kingdom

**DOI:** 10.3390/v15030721

**Published:** 2023-03-10

**Authors:** Joshua D. Jones, Clare Trippett, Mehrunisha Suleman, Martha R. J. Clokie, Jason R. Clark

**Affiliations:** 1 UK Phage Therapy, 272 Bath Street, Glasgow G2 4JR, UK; 2 CPI, 1 Union Square, Central Park, Darlington DL1 1GL, UK; 3 The Ethox Centre, University of Oxford, Li Ka Shing Centre for Health Information and Discovery, Old Road Campus, Oxford OX3 7LF, UK; 4 Department of Genetics and Genome Biology, University of Leicester, Leicester LE1 7RH, UK; 5 Fixed Phage, West of Scotland Science Park, Block 2, Kelvin Campus, 2317 Maryhill Road, Glasgow G20 0SP, UK

**Keywords:** bacteriophage, phage therapy, United Kingdom

## Abstract

Bacteriophage (phage) therapy is a promising alternative antimicrobial strategy with the potential to transform the way bacterial infections are treated. In the United Kingdom, phages are classed as a biological medicine. Although no phages are licensed for UK use, they may be used as unlicensed medicinal products where licensed alternatives cannot meet a patient’s clinical needs. In the last 2 years, 12 patients in the UK have received phage therapy, and there is burgeoning clinical interest. Currently, clinical phage provision in the UK is ad hoc and relies upon networking with international sources of phages. The provision of phage therapy in the UK will not progress beyond an increasing number of ad hoc cases until an onshore sustainable and scalable source of well-characterised phages manufactured in accordance with Good Manufacturing Practice (GMP) is established. Here, we present an exciting new collaboration between UK Phage Therapy, the Centre for Phage Research at University of Leicester, CPI, and Fixed Phage. These partners, and others as we develop, will establish sustainable, scalable, and equitable phage therapy provision in the UK. We set out a vision for how phage therapy will be integrated into the NHS and healthcare more broadly, including the complementarity between licensed (cocktail) and unlicensed (personalised) phage preparations. Key elements of phage therapy infrastructure in the UK will be GMP phage manufacturing, a national phage library, and a national clinical phage centre. Together, this infrastructure will support NHS microbiology departments to develop and oversee phage therapy provision across the UK. As it will take time to deliver this, we also describe considerations for clinicians seeking to use unlicensed phage therapy in the interim. In summary, this review sets out a roadmap for the delivery of clinical phage therapy to the UK, the benefits of which we hope will reverberate for patients for decades to come.

## 1. Background

Bacteriophages (phages) are naturally occurring viruses that infect bacteria in a generally species-, and sometimes even strain-, specific manner. Phages are the most abundant biological entity on the Earth. Found wherever bacteria are, phages also form a significant part of the human commensal microbiota [1]. We are, and have evolved in, constant exposure to phages. Phages were first used to treat bacterial infection in 1919 [2]. This heralded a ‘golden era’ for phage therapy in the 1920s and 1930s, which saw phages used widely. Interest in phage therapy declined due to a combination of factors. Importantly, the mass production of antibiotics in the 1940s sealed the fate of phage therapy as antibiotics were easier to make, market, and use. This decline in phage therapy was not universal, and phages continued, and continue, to be used in the geopolitical East, particularly Russia, Georgia, and Poland [3]. Now, over 100 years since phage therapy was first used, the antibiotic resistance crisis is driving a global renaissance in phage therapy. Antibiotic resistance is an urgent global health issue, and was associated with an estimated 4.95 million deaths worldwide in 2019 [4]. Alternative antimicrobials that reduce our use of and reliance upon antibiotics are therefore urgently needed.

Phage therapy is an exciting antimicrobial strategy with the potential to transform the care of bacterial infections. Simple phage suspensions can be delivered in two formats: pre-formulated off-the-shelf cocktails or personalised phage cocktails, the latter informed by screening of a bacterial pathogen against a library of phages. Clinical and safety trials of phage therapy, administered by a variety of routes, have consistently demonstrated safety [5,6,7,8,9,10,11,12,13,14,15,16,17,18]. Moreover, a recent systematic review of observational clinical data from 2241 patients treated with phages since the year 2000 found that phage therapy was well tolerated, with any adverse events deemed mild [19]. To the best of our knowledge, there have only been 12 instances of possible clinical adverse effects to modern phage therapy, none of which were believed by the report authors to be directly attributable to phages [20,21,22,23,24,25,26,27,28]. Reassuringly, there have also been multiple efficacious and side effect-free reports of phage therapy by invasive routes of administration (e.g., intravenous, intra-articular) and in immunocompromised patients [29,30,31,32,33,34]. In contrast to phages, and clearly within the context of antibiotics being widely used, it is worth noting that antibiotic-associated adverse drug events are comparably common; one 2017 study reported that 20% of patients had antibiotic-associated adverse drug events [35]. Together, all the evidence to date paints a reassuring picture of the safety of phage therapy, consistent with our exposure to, and co-evolution with, phages. Perhaps unsurprisingly, given the safety profile of phages relative to antibiotics, some patients have even suggested that, if given the choice, they would prefer to try phage therapy before intravenous antibiotics [36].

There is also a significant body of data to support the efficacy of phage therapy. A recent systematic review of clinical data found that 79% of 1904 phage-treated patients saw clinical improvement and 87% of 1461 patients achieved bacterial eradication [19]. These data are particularly compelling given that most of these patients had infections refractory to antibiotics, but which resolved with phage treatment. However, there is an explainable discrepancy between the efficacy of phage therapy reported by observational and clinical trial data. This is because it is difficult for trials to consistently achieve the “goldilocks constellation” of getting a sufficient quantity of phages with the correct specificity to the site of infection; an in-depth exploration of trial data has been published elsewhere [37]. Nevertheless, the Antibiotic Resistance Leadership Group in the United States and Healthcare Improvement Scotland have concluded that there is sufficient evidence to consider phage therapy as a treatment option in patients with difficult-to-treat infections [38,39].

Phage therapy has several key advantages. Relative to antibiotics, phages are quick and inexpensive to produce. Although resistance to phages can develop, the evolutionary cost can be high, and if correct phage formulations are used, resistance rates can be very low [40]. Furthermore, the diversity of phages is such that new phages to which the bacteria are not resistant can either be found or phages can be “trained” on the bacteria [41]. Moreover, some combinations of phages and antibiotics can be synergistic, putting the bacteria under evolutionary pressure from two angles, with resistance to either antibiotics or phages potentially rendering the bacteria more susceptible to the other [42]. Phages can also be used to address antibiotic tolerance, a problem arguably larger than antibiotic resistance and thought to underpin many chronic infections which remain, on paper, sensitive to antibiotics. Unlike antibiotic resistance, tolerance to antibiotics can be afforded by biofilm production or phenotypic changes in bacterial cells. Some phages degrade biofilms and enter dormant “persister” bacterial cells, ready to replicate when those cells themselves become metabolically active [43,44,45]. A further advantage of phage therapy is that because phages are so specific, they are considered to leave commensal flora intact, reducing the likelihood of opportunistic infections [46]. There are also no reports of allergic responses to phages, making them suitable alternatives for patients with antibiotic hypersensitivity [47].

The United States, Australia, France, Germany, and Belgium are among the Western nations using phage therapy for difficult-to-treat infections. So far, in the UK, one burn-wound patient received phage therapy in 2006 and there was a phase I/II clinical trial of phages for chronic otitis in 2009 [5,48]. The use of phage therapy has increased recently, and in the last 4 years, 2 cystic fibrosis patients at Great Ormond Street Hospital (London, UK) and 10 diabetic foot infection patients in 2 Scottish hospitals have received phage therapy [49] (Young and colleagues, in submission, 2023). To date, phage therapy in the UK has been held back by lack of sustainable access to phages manufactured according to the internationally recognised quality standard of Good Manufacturing Practice (GMP) and the misconception that current regulations are not appropriate. This paper outlines how an exciting consortium will make phage therapy possible for the National Health Service (NHS) by marrying world-leading phage researchers and Good Manufacturing Practice expertise with an exciting and tangible vision for phage therapy in the UK.

## 2. The Regulatory Status of Phage Therapy in the UK

There have been misconceptions about the regulations around phage therapy in the UK. A major misconception is that the unique nature of phages means that new regulations are required to support phage use. This is not the case, as there are no regulatory barriers to the appropriate use of phage therapy in the UK. However, as phage therapy progresses, there may be need for regulatory refinement.

Naturally occurring phages, i.e., not designated as genetically modified, are classified by the UK medicines regulator, the Medicine and Healthcare products Regulatory Agency (MHRA), as a biological medicine, not an Advanced Therapeutic Medicinal Product [50]. Although there are no licensed phage products in the UK, phages may be used in certain circumstances as an unlicensed medicinal product, also known as a “special” [50,51]. Specials can be used in accordance with MHRA guidance on an unlicensed (sometimes referred to as “named-patient”) basis when a clinician determines that licensed alternatives (e.g., antibiotics) are not fulfilling a patient’s clinical needs [51]. Phages imported for use as an unlicensed medicine do not need to be manufactured according to GMP, while phages manufactured in the UK must be produced to GMP by the holder of a manufacturer’s specials license, regardless of volume [51]. This means it is not permitted for UK non-GMP laboratories to manufacture small volumes of phages for clinical use on a per-patient basis. It is not clear whether phages discovered in the UK can be exported for non-GMP manufacturing before re-importation, but this may appear to not be within the spirit of the regulations. Only appropriately licensed bodies can import phages and importations must be undertaken following MHRA guidance. For UK-based clinical trials, phages must be manufactured to GMP [52]. Phages which are being trialled are known as investigational medicinal products (IMPs). Notably, “compassionate use” is the clinical use of an IMP that has entered the marketing-authorisation application process, whereas “named-patient” means clinical use of an unlicensed medicine (special) that is not in clinical trials [51,53,54].

The UK’s NHS is divided into geographically distinct health authorities responsible for healthcare provision in their area. These are referred to by different terms depending on where they are in the UK, but for the purposes of this article, all NHS authorities will be referred to as Trusts. All NHS Trusts can and do use unlicensed medicines. Phages are no different than other unlicensed medicines and existing unlicensed medicines policies therefore provide appropriate clinical governance for phage therapy. When phages are used as part of clinical care in this way, it is the existing unlicensed medicines policy that provides governance, not research governance mechanisms such as ethics and “R&D” approval [55].

Patients for whom unlicensed phage therapy may be appropriate include those with antibiotic resistant infections; antibiotic susceptible but clinically recalcitrant chronic infections; reasonably foreseen acute risk to life or limb despite appropriate antibiotic treatment; other patient-specific factors that preclude the use of appropriate antibiotics (e.g., renal failure, allergy, drug–drug interactions, or intolerable side effects); or cases where further medical intervention is preferred to surgery (e.g., high-risk surgical candidate). It is important to document the rationale for phage therapy (i.e., why licensed alternatives were not suitable); input from a multi-disciplinary team, or the opinions of clinical colleagues, may be useful.

## 3. The Current State of Phage Therapy Provision in the UK

Currently, unlicensed medicines policies (sometimes termed non-formulary requests) can support clinicians wanting to access phage therapy for patients whose clinical needs cannot be met by antibiotics. However, sourcing high-quality phages is challenging, especially for those unfamiliar with phages. Access to phages is currently on an ad hoc basis and driven largely by networking between clinicians and clinical phage laboratories. This means that access to phage therapy is not currently equitable, with large variations in clinician awareness and NHS capacity to deliver phages as an unlicensed medicine across the UK. There have been some international initiatives to “join up” clinical demand with collections of phages. For example, Phage Directory has successfully connected clinicians seeking phages to groups with appropriate phages [56]. However, fundamentally, these disparate sources are not sustainable, scalable, or of a consistent quality. Phages can be contract manufactured according to GMP, but at a cost of over GBP 500,000 for phages sufficient for a few hundred patients, this is also not sustainable or scalable. The provision of phage therapy in the UK will not progress beyond an increasing number of ad hoc cases until an onshore sustainable and scalable source of GMP phages is established. Sustainable and scalable access to GMP phages is similarly curtailing the expansion of phage therapy in other countries. Notably, in countries where phage therapy has progressed, clinically and/or commercially, this has typically been underpinned in some form by public funding. We hope that the UK Government and ongoing Parliamentary Science and Technology Select Committee Inquiry will recognise the potential of public funding to simultaneously transform the clinical, research, and commercial phage therapy landscapes in the UK. Access to well-characterised GMP phages would facilitate multiple phage therapy clinical trials in the UK and catalyse UK phage businesses. We therefore outline in the following sections how we are actively working to deliver a well-characterised, sustainable, equitable, and scalable source of GMP phages that will transform the way we treat infection in the UK.

## 4. The Future of Clinical Phage Therapy in the UK

**Figure 1:**
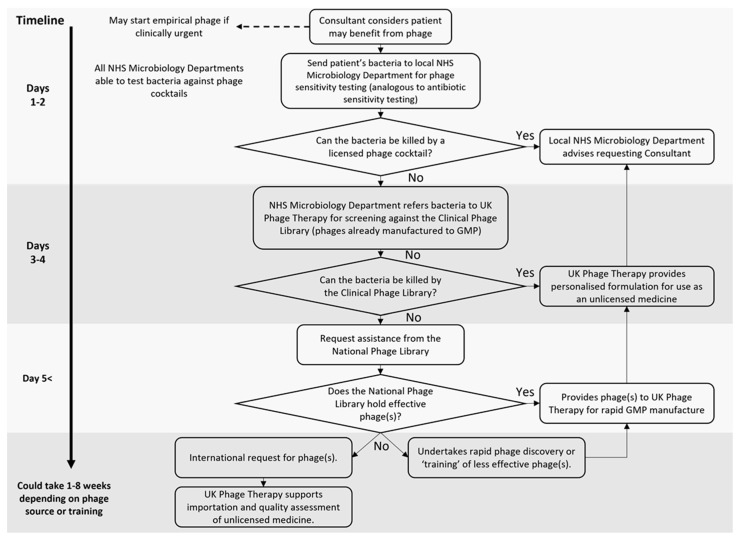
Future delivery of phage therapy by NHS microbiology services, UK Phage Therapy, and a National Phage Library.

Phage therapy should be integrated into all stages of care in the NHS. Figure 1 shows how local existing NHS microbiology services, UK Phage Therapy, and a National Phage Library will work together to deliver phage therapy in the future. Hospital consultants will have access to licensed off-the-shelf phage cocktails, produced in accordance with GMP, which cover a broad range of common bacterial pathogens. As licensed medicines, these will be procured by the NHS in the usual manner. In contrast to unlicensed use, licensed cocktails may be used to treat infections much earlier, helping reduce the numbers of patients progressing to more advanced stages of disease. Just as for antibiotics, all hospitals will be able to hold stocks of these off-the-shelf cocktails. While it is crucial that the sensitivity of a patient’s bacteria to the phages is demonstrated, in cases of clinical urgency or when a clinical isolate cannot be obtained, it is conceivable that such cocktails may be given empirically, most likely but not necessarily alongside antibiotics.

Consistent with current practice, local NHS microbiology departments will be responsible for diagnosing the bacterial pathogen(s) responsible and testing their sensitivity to antibiotics. It typically takes microbiology departments 1–2 days to report antibiotic sensitivity results. In the future, microbiology departments will also be able to simultaneously test the susceptibility of the pathogen(s) to the off-the-shelf phage cocktail(s). Like antibiotic sensitivity testing, bacterial growth is the rate limiting step in obtaining phage sensitivity results. Consequently, the turnaround time for phage sensitivity results would be expected to be similar to that for current antibiotic sensitivity testing, around 1–2 days. Diagnostics in NHS microbiology departments is initially expected to rely on classical microbiological techniques with scalable approaches, such as the Omnilog platform [57], adopted where this is cost-effective.

It is envisaged that due to the broad spectrum of pathogens covered, off-the-shelf cocktails will meet the majority of patient needs. Should a patient’s bacteria either not be susceptible to or not be within the target range of an off-the-shelf cocktail, then the NHS microbiology department will refer the isolate to UK Phage Therapy. As the UK’s clinical phage centre, UK Phage Therapy will hold a library of phages manufactured to GMP known as the Clinical Phage Library. UK Phage Therapy will screen the patient’s isolate against the Clinical Phage Library and prepare a personalised phage cocktail which will be sent back to the patient’s own Trust for use on an unlicensed basis. The use of this cocktail will then be overseen by the local NHS microbiology department.

If suitable phages cannot be found in the Clinical Phage Library, UK Phage Therapy will request support from the National Phage Library, a large collection of well-characterised phages hosted at the University of Leicester’s Centre for Phage Research. If suitable phages are still not identified, then either novel naturally occurring phages will be sought from environmental sources or existing phages will be “trained” by in vitro directed evolution. In cases of clinical urgency, streamlined characterisation will be undertaken before expedited GMP production. Alternatively, UK Phage Therapy may seek phages from international phage libraries. Together, this system represents a cohesive infrastructure to sustainably deliver phage therapy as part of hospital care in the NHS.

The initial focus of UK Phage Therapy will be on the provision of simple suspensions of phages for the treatment of infections. Such simple suspensions are clinically very flexible as they are suitable for administration by a variety of routes. Moreover, the administration of phage suspensions is practically straightforward and does not require any specialist clinical skills not already available in all Trusts, facilitating the delivery of equitable phage provision across the UK. Patients should therefore receive phage therapy in the Trust in which they would have already been receiving care for their infection [58].

Such is the simplicity of phage administration for some indications, such as chronic wounds, that phage therapy could readily be administered on an outpatient basis or even in general practice or community nursing settings. The integration of phage therapy at these levels of care will be advantageous for the NHS, alleviating the burden on secondary care, and for patients in rural settings. While simple phage suspensions will cover a broad range of clinical needs, as phage therapy advances, needs will emerge for more specialised or complex formulations. However, it is anticipated that there will be a dichotomy between phage products used either for the treatment or prevention. While phage suspensions or alternative formulations that can be used flexibly in clinical settings will play a key role in the treatment of infections, it is likely that more complex commercial phage products, such as wound dressings, will play a key role in the prevention of infection. Intellectual property protection of naturally occurring phages alone is difficult, if not impossible [59]. However, this is unlikely to pose a barrier to the development and licensing of phage therapeutics, with the production of simple phage suspensions conceptualised as analogous to generic drug manufacturing. Aside from phage-coated dressings, there is also interest in phage-coated implants or catheters, all of which are commercially attractive settings and ones in which infection prevention is paramount [60,61]. This vision is therefore not just for the integration of phage therapy into the NHS, but one which sees the full potential of phage therapy exploited at all levels of healthcare, from over-the-counter products to specialist hospital care. Although briefly acknowledged in the UK Government’s current 5-year national action plan for tackling antimicrobial resistance [62], achieving this vision will require recognition of phage therapy as a key part of the UK’s response to antimicrobial resistance, with the potential to both reduce antibiotic use and provide a means to treat patients with antibiotic refractory infections. Delivering this vision will represent a historical transformation in the way that we treat infections in the UK.

## 5. Delivering Phage Therapy for the United Kingdom

**Figure 2:**
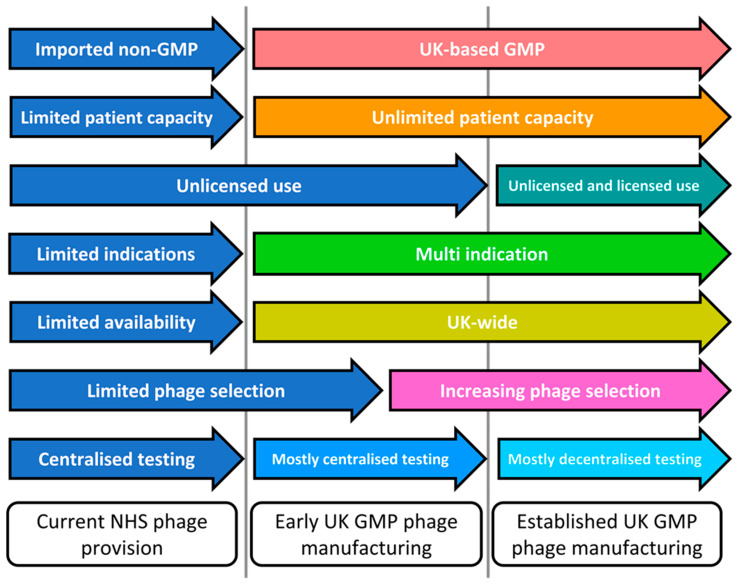
The current and future stages of UK phage therapy infrastructure.

Figure 2 shows the current and future stages of UK phage therapy infrastructure. A recent Scottish initiative, led by the first author, saw 1 type of anti-staphylococcal phage used as a topical adjunct in 10 patients with difficult-to-treat diabetic foot infections, and illustrates the current limitations faced by phage therapy in the UK (Young and colleagues, in submission, 2023). This initiative had a limited capacity for patients with a single type of indication and had access to only one type of phage, meaning nothing could be done for patients whose isolates were not susceptible to the phage. Clinical phage provision in the NHS is ongoing but will still rely on unsustainable sources of non-GMP phages. Fundamentally, phage therapy will not progress in the UK without sustainable onshore access to GMP phages.

## 6. The National Microbiome and Phage Bioprocess Innovation Centre: A Sustainable Source of GMP Phages

We intend to take a two-stage approach to delivering sustainable GMP phage provision for the UK (Figure 2). CPI is an independent technology innovation centre and founding member of the High Value Manufacturing Catapult [63]. Working with Fixed Phage, a company developing commercial phage products using their patented phage-stabilising technology, and UK Phage Therapy, CPI plans to develop GMP manufacturing capabilities for both phages and microbiome therapeutics. As shown in Figure 2, early UK GMP phage provision will develop into a source that could respond to unsolicited requests from the NHS. Initially, a small number of phages will be manufactured to GMP, precluding the provision of personalised phage cocktails, a capability that will develop as the GMP Clinical Phage Library expands. However, we anticipate that the volumes of phages manufactured would be sufficient that there will not be any limit on the number of requests that could be fulfilled. Once GMP phages are available, clinical isolates will initially be sent to UK Phage Therapy for centralised phage susceptibility testing, but it is anticipated that NHS centres will progressively be trained to do their own testing. This will involve the creation of clinical phage therapy as a new sub-specialty within clinical microbiology. Communication with all sectors of the NHS about clinical phage therapy as a new sub-specialty, including with primary care and district general hospitals, will be vital and will take place through existing professional clinical and managerial channels. A key role of UK Phage Therapy, supported by the National Phage Library, will be capacity building within NHS microbiology departments across the UK, preparing them to undertake testing locally. Extensive patient and public engagement will also be needed, ensuring that the message about phage therapy and phages as “good viruses” is clearly communicated.

Small-scale GMP manufacturing will bridge phage provision in the UK until permanent GMP manufacturing facilities are established as part of an intended national Microbiome and Phage Bioprocess Innovation Centre. The centre is planned for development through public/private partnerships and will provide a unique facility that will act as a springboard for translating microbiome and phage research and development in the UK, underpinned by world-class GMP manufacturing facilities to accelerate innovation in microbiome-based healthcare solutions. UK Phage Therapy will use these facilities to deliver national clinical phage provision and put off-the-shelf suspensions of phages through clinical trials. We envisage a limited range of cost-effective ‘national’ phage cocktails which will be subject to regular reformulation to keep pace with changing microbial ecology. Minimal, if any, regulatory adjustment will be required to enable this. As licensed medicines, phage cocktails will be available to, and funded by, the NHS via the usual procurement routes, while personalised phage preparations will be used on an unlicensed basis. Through partnering with the Microbiome and Phage Bioprocess Innovation Centre, UK Phage Therapy will be the UK’s specialist clinical phage centre and deliver sustainable and scalable high-quality phage therapy for the UK.

## 7. The National Phage Library

The Centre for Phage Research at the University of Leicester plans to host a National Phage Library, which will draw on phage sources from across the country to deliver a world-leading phage biobank. As shown in Figure 1, the National Phage Library will play a key role in underpinning robust and sustainable phage provision in the UK. It is expected that as a resource the National Phage Library will also be of interest to academics and businesses with broader interests in phage applications.

From the perspective of phage production in the UK, the first phages to be manufactured to GMP will be phages previously used for phage therapy elsewhere and for which permission for UK manufacture has been obtained. In addition to this, the UK will establish its own range of therapeutic phages, liaising with other resources where needed. The National Phage Library and the infrastructure and expertise at the Centre for Phage Research will play a key role in this, providing and coordinating the fundamental academic underpinning that will make sustainable phage therapy possible for the UK.

The already substantial and well-characterised phage collection at Leicester’s Centre for Phage Research will be expanded in a targeted programme of systematic clinically-orientated phage discovery. In addition to phages that will come from colleagues throughout the UK, new phages will be isolated from environmental and clinical reservoirs. Each phage will have a unique library record containing key information relevant for clinical characterisation, such as key classical microbiological parameters and genome sequences with confirmation of the absence of genes associated with lysogeny, toxins, or antibiotic resistance. The Centre for Phage Research will also work with structural biologists to determine a mechanistic understanding of key phages and with other key academic entities such as the Leicester Institute for Advanced Studies to ensure the cultural landscape surrounding phage usage is conducive. As a focal point for academic phage therapy, the National Phage Library will streamline interactions between UK Phage Therapy and the academic community and help coordinate a national programme of clinically relevant research.

## 8. UK Phage Therapy: The National Clinical Phage Centre

As the national clinical phage centre, UK Phage Therapy will work closely with the Microbiome and Phage Bioprocess Innovation Centre, academic partners in the National Phage Library, NHS Trusts, and businesses. UK Phage Therapy will have three main objectives. As described above, UK Phage Therapy will make GMP phages readily available for patient care in the NHS. This will be achieved by first enabling sustainable use of high-quality phages in an unlicensed capacity and subsequently the development of off-the-shelf phage cocktails alongside which unlicensed, personalised phage therapy will continue. Supplying GMP phages internationally will also be considered. Second, UK Phage Therapy will engage in applied clinical phage research at the intersection of clinical, commercial, and academic sectors. Areas of research and development will include sustainable and scalable phage diagnostics for the NHS, working with businesses to drive clinically useful commercial ideas and providing expertise to support the delivery of phage clinical trials. The third objective of UK Phage Therapy is to drive capacity building in the NHS and provide a focal point and leadership for clinical phage therapy in the UK.

## 9. The Role of the NHS

The NHS will be central to the delivery of phage therapy at scale. The provision of phage therapy represents a laboratory-based microbiology service. The use of existing microbiological capabilities in NHS Trusts will create a disseminated organisation of phage therapy in the UK and means that there will not be a need for the creation of specialist phage centres for each of the medical specialities. This is in line with conceptually considering phages as we would a new antibiotic, i.e., a treatment equally available throughout the NHS. The distribution of phage expertise into every Trust will benefit patients by creating an equitable system with minimal delays to accessing phage therapy. However, clinicians within specialties are likely to want to liaise with each other to share their experiences of using phage therapy and develop clinical practice. Established specialty-specific forums are likely to provide the most suitable setting for such discussions.

## 10. The Rationale

This approach will deliver sustainable, scalable, and cost-effective phage therapy to the NHS, backed by a wealth of well-characterised phages that are safe for therapeutic use in humans. As a patient-centred approach, this will initially prioritise access to unlicensed phages manufactured to GMP in the UK. This will have a significant positive impact on the care of these patients. Notably, phage therapy will to some extent always be used as an unlicensed medicine and unlicensed use of phage therapy will therefore precede licensed use, which will be achieved at scale following clinical trials of off-the-shelf phage cocktails. Considering unlicensed phage therapy in the same way as other unlicensed medicines, it is appropriate for NHS Trusts to each take responsibility for their own use of unlicensed phages in accordance with well-established local unlicensed medicines policies. We note that because of the organisation of the NHS into discrete Trusts, a single, standardised ‘pathway’ for delivering unlicensed phage therapy is not required and is at any rate unlikely.

The approach outlined here is specific to the UK and it is important to recognise the value of taking an informed national approach underpinned by dialogue with the appropriate regulator. What works in Australia or Belgium may not work in other countries and conversely what works in the UK within our health system may not be suitable for other contexts. We do not expect that the clinical, manufacturing, or technical models developed in the UK will necessarily be transferable. While there is value in observing the approaches of others, the success of phage therapy globally will ultimately reflect the success of distinct approaches taken by individual countries.

## 11. Phage Therapy Today

We understand that many clinicians, and patients, reading this will want phage therapy for immediate clinical problems. As described above, there are no regulatory barriers to appropriate phage therapy use as an unlicensed medicine. However, phage therapy provision to individual patients is presently very labour-intensive. If sporadic patient needs could be met in more Trusts, there would be substantial benefit to individual patients, but it would not create an equitable or cohesive system that will benefit thousands of patients. Thus, although the appropriate provision of phages to individual patients is desirable, we are seeking to take an ethically pragmatic approach and balance our resources between meeting immediate patient needs and the systematic introduction of phage therapy into the NHS.

Where provision for individual patients is undertaken, this must be done with care and in accordance with relevant local policies and legislation. Assessing the quality of phages is likely to be challenging for NHS staff unfamiliar with phages and the use of impure phage preparations could yield adverse effects detrimental to the wider field. Similarly, care must be taken to ensure the importation of phages is undertaken according to MHRA guidance [51], with queries referred to the MHRA. Clinically, the use of phage therapy must be undertaken in an appropriately evidence-based manner, with the rationale and supporting literature stated in any unlicensed medicines documentation. Successful phage therapy relies on getting the right phage(s) to the right place to treat an infection containing sufficient susceptible bacterial hosts. This constellation of factors can vary widely between patients, even those with seemingly similar infections. One-size-fits-all ‘standard operating procedures’ are therefore not currently appropriate. While it may be that in several years clinical protocols with due flexibility built-in can be devised for specific indications, retaining the flexibility for tailoring both the phages and clinical approach on a per-patient basis will be vital for the long-term success of phage therapy.

It is similarly important to ensure that procedures for microbiological diagnostics follow standard practice and guidelines, with any bacteriological diagnostics being carried out by the appropriate NHS department. While academics and others may be keen to support the use of phage therapy, data protection must be a priority and no patient information or identifiable details about a patient’s case should leave the NHS; local Caldicott Guardians and/or information governance staff should be consulted if uncertainty arises. A cornerstone of the provision of unlicensed medicines in the UK is that any such requests should be unsolicited [51]. This highlights the care that needs to be taken by those not involved in patient care to ensure that they are not soliciting the use of phage therapy.

Caution should also be exercised around data collection. Health Research Authority guidance emphasises the importance of generalisability and transferability, and a scientifically sound method, in defining research [64]. In contrast, clinical case data do not use a scientific method or predetermined endpoints and any observations would not be generalisable or transferable. While it is sensible to collect any data about a patient that is needed for their immediate or ongoing care and for that to be made available in case reports, the collection of data beyond that required for the care of the patient risks becoming an unauthorised clinical trial, even if only one patient is treated [65]. The extent of data collection permitted in other Western contexts cannot be taken as a proxy for what is acceptable to the UK authorities. The use of phages in an unlicensed capacity in the UK must reflect a response to genuine individual clinical needs and this is at odds with prospective protocol-driven approaches. For example, the Australian standardised treatment and monitoring protocol is a clinical trial [66], and similar approaches in the UK are likely to also fall under the remit of a clinical trial. It is also conceivable that clinicians may attempt to collect research data from clinical cases in alternative ways, e.g., by ensuring all phage patients are enrolled into ongoing observational studies from which their data could then be isolated. Such approaches to data collection from the use of unlicensed medicine are inappropriate and should not be used to circumvent clinical trials for research data collection. The regulators should be consulted if there is uncertainty about whether a planned activity is a clinical trial. It is also worth noting that, as an unlicensed medicine, phages cannot be used instead of antibiotics to reduce antibiotic use or to save money; nor can phages be used for clinical convenience, e.g., the decolonisation of a medical device where it could otherwise easily be replaced.

## 12. Conclusions

Phage therapy fits within the existing regulatory framework in the UK and there are presently no regulatory barriers to appropriate phage use or development; however, as phage therapy progresses, there may be need for regulatory refinement. Phage therapy in the UK will not progress beyond ad hoc clinical cases without access to sustainable and scalable GMP phage production. An exciting collaboration, initially between UK Phage Therapy, CPI, Fixed Phage, and the Centre for Phage Research at the University of Leicester, is working to deliver this key phage therapy infrastructure for the UK. We envisage that as we grow, and as the antimicrobial resistance crisis continues to loom, we will attract a wide range of partners. UK Phage Therapy will be the UK’s clinical phage centre, combining access to GMP phages with a clinical phage screening service. The long-term vision is that the clinical needs of most patients will be met by off-the-shelf cocktails, with a minority requiring specialist-personalised phage therapy. While it may be possible to support small numbers of patient requests in the interim, we are primarily directing our efforts towards delivering this vision which will ultimately benefit thousands of patients and transform the UK’s approach to infection medicine.

## Data Availability

Data sharing is not applicable to this article as no datasets were generated or analysed during the current study.

## Abbreviations

GMP, Good Manufacturing Practice; MHRA, Medicines and Healthcare Products Regulatory Agency; NHS, National Health Service.
